# The C-terminal domains of ADAMTS1 contain exosites involved in its proteoglycanase activity

**DOI:** 10.1016/j.jbc.2023.103048

**Published:** 2023-02-21

**Authors:** Alexander Frederick Minns, Yawei Qi, Kazuhiro Yamamoto, Karen Lee, Josefin Ahnström, Salvatore Santamaria

**Affiliations:** 1 Department of Biochemical Sciences, School of Biosciences, Faculty of Health and Medical Sciences, University of Surrey, Guildford, Surrey, United Kingdom; 2 Department of Immunology and Inflammation, Imperial College London, London, United Kingdom; 3 Institute of Life Course and Medical Sciences, University of Liverpool, Liverpool, United Kingdom

**Keywords:** *ADAMTS*, *proteoglycan*, *versican*, *aggrecan*, *proteolysis*, *exosite*

## Abstract

A disintegrin-like and metalloproteinase with thrombospondin type 1 motifs (ADAMTS1) is a protease involved in fertilization, cancer, cardiovascular development, and thoracic aneurysms. Proteoglycans such as versican and aggrecan have been identified as ADAMTS1 substrates, and *Adamts1* ablation in mice typically results in versican accumulation; however, previous qualitative studies have suggested that ADAMTS1 proteoglycanase activity is weaker than that of other family members such as ADAMTS4 and ADAMTS5. Here, we investigated the functional determinants of ADAMTS1 proteoglycanase activity. We found that ADAMTS1 versicanase activity is approximately 1000-fold lower than ADAMTS5 and 50-fold lower than ADAMTS4 with a kinetic constant (*k*_cat_/*K*_m_) of 3.6 × 10^3^ M^−1^ s^−1^ against full-length versican. Studies on domain-deletion variants identified the spacer and cysteine-rich domains as major determinants of ADAMTS1 versicanase activity. Additionally, we confirmed that these C-terminal domains are involved in the proteolysis of aggrecan as well as biglycan, a small leucine-rich proteoglycan. Glutamine scanning mutagenesis of exposed positively charged residues on the spacer domain loops and loop substitution with ADAMTS4 identified clusters of substrate-binding residues (exosites) in β3-β4 (R756Q/R759Q/R762Q), β9-β10 (residues 828–835), and β6-β7 (K795Q) loops. This study provides a mechanistic foundation for understanding the interactions between ADAMTS1 and its proteoglycan substrates and paves the way for development of selective exosite modulators of ADAMTS1 proteoglycanase activity.

A disintegrin-like and metalloproteinase with thrombospondin type 1 motifs (ADAMTS1) was initially identified in 1997 as a protein highly expressed in the murine colon 26 adenocarcinoma cell line (1) and was the first member of the ADAMTS family to be identified. This family has grown since then and now includes 19 genes in humans (2). Transcriptomics analysis of 55 tissue types showed that ADAMTS1 was predominantly expressed in ovary, placenta, adipose tissue, smooth muscles, fallopian tubes, and urinary bladder. Single cell RNA-seq further identified smooth muscle cells, adipocytes, endothelial cells, fibroblasts, extravillous trophoblasts, endometrial stromal cells, Sertoli cells, and cardiomyocytes as the major ADAMTS1-expressing cell types (https://www.proteinatlas.org/) (3). As is highlighted by its expression pattern, ADAMTS1 plays a role in a wide range of biological processes, including fertility and development of the cardiovascular and urogenital systems (4). *Adamts1* KO mice exhibited significant postnatal lethality due to urological defects (5). Other phenotypes included growth retardation (6), female subfertility due to impaired ovulation (6, 7, 8, 9, 10), and smaller arterial and venous lumens (11). ADAMTS1 is also involved in endocardial valve maturation and myocardial trabeculation (12, 13). There is presently conflicting evidence on the role of ADAMTS1 in cardiovascular pathologies (14). ADAMTS1 may contribute to atherosclerosis, possibly by enhancing cell proliferation and migration (15), whilst knockout of *Adamts1* in mice resulted in either reduced or increased incidence and mortality of thoracic aortic aneurysms dependent on the specific model and/or mouse strain used (16, 17). Other studies have also suggested that ADAMTS1 plays both protumorigenic and antitumorigenic roles in cancer (reviewed in ref. (18)), depending on whether its expression is dysregulated in tumor cells or the surrounding stroma (19). These studies underscored a complex patho/physiological function of ADAMTS1. However, to date, what role the proteolytic activity of ADAMTS1 plays in these processes is unknown.

On the basis of sequence homology, ADAMTS1 has been assigned to a subgroup of ADAMTS proteases comprising so called proteoglycanases (ADAMTS4, 5, 8, 9, 15, and 20), whose members have been reported to share the ability to cleave chondroitin sulfate proteoglycans (20) (although we have recently shown that ADAMTS8 shows negligible proteoglycanase activity *in vitro* (21)). Proteoglycans are highly decorated with glycosaminoglycans (GAGs) and represent major structural components of the extracellular matrix (ECM). By generating Donnan osmotic pressure, GAGs contribute to the biomechanical properties of the ECM. Through their interactions with cytokines, growth factors, proteases, and inhibitors (22), GAGs also modulate tissue response to external stimuli. ADAMTS1 has been shown to cleave the proteoglycans aggrecan (23) and versican (24) as well as biglycan (21). Aggrecan is the major proteoglycan in cartilage, but it is also expressed in cardiovascular tissues, where the expression of versican is predominant (14, 25). In aggrecan, ADAMTS1 has been reported to cleave at the Glu^2053^-Leu^2054^ (23), Glu^1679^-Gly^1680^, and Asn^360^-Phe^361^ (26) bonds (human aggrecan numbering, UniProt ID: P16112-1). While cleavage at the ‘canonical’ aggrecanase site, Glu^392^-Ala^393^, has been reported (26), we have failed to detect consistent cleavage at this site even at high ADAMTS1 concentrations (500 nM) (21), suggesting that cleavage in this region, crucial for aggrecan structure and function (27), is very inefficient. These findings and the observation that *Adamts1* KO mice did not exhibit decreased aggrecan turnover, nor were protected in an inflammatory model of cartilage degradation (28), strongly indicate that aggrecan may not be a physiologically relevant ADAMTS1 substrate. In contrast, *Adamts1* KO resulted in decreased versican proteolysis and versican accumulation (4). Versican is present in five isoforms, resulting from alternative splicing of exons 7 and 8, which encode the central, GAG-rich domains called αGAG and βGAG, respectively (29). These isoforms all retain the globular G1 and G3 domains at their N- and C- termini, respectively (29). Isoforms V0, V1, and V3 are more ubiquitously expressed. The first described cleavage event by ADAMTS1 occurs at Glu^441^-Ala^442^ in the central βGAG domain of the V1 isoform, generating an N-terminal fragment called versikine (24), while we recently reported three additional cleavage sites upstream this ‘canonical’ versicanase site (Glu^768^-Leu^769^, Glu^923^-Arg^924^, and Gln^1027^-Leu^1028^, UniProt ID: P13611-2) (30).

Overall, the characterization of ADAMTS1 proteoglycanase activity has been limited, mainly due to a lack of quantitative assays to measure generation of cleavage products and, consequently, determine kinetic parameters. We have recently described an ELISA based on the specific recognition of versikine by a neoepitope antibody (31). Since neoepitope antibodies do not recognize the uncleaved protein, they represent the ideal tool to investigate cleavage of full-length (FL) proteoglycans at specific sites. Here, by coupling classic enzyme kinetics with versikine ELISA, we aimed to identify the residues distant from the active site (*i.e.*, exosites) used by ADAMTS1 to recognize and cleave versican. Using a series of variants, we show that ADAMTS1 requires its C-terminal domains for versicanase activity and identified the residues involved. Additionally, we showed that similar structural determinants are required for cleavage of other proteoglycans, such as aggrecan and biglycan.

## Results

### Expression and purification of recombinant ADAMTS1 and its domain-deletion variants

**Figure 1:**
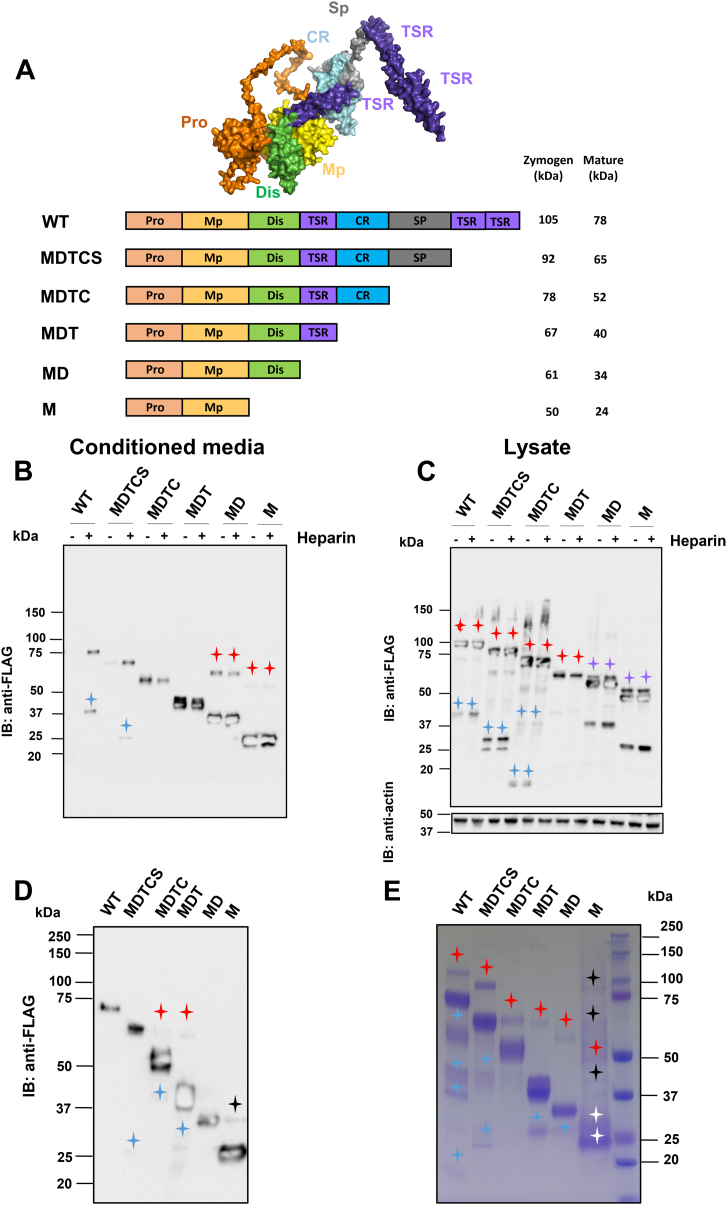
**Expression and purification of ADAMTS1 domain-deletion variants**. *A*, AlphaFold model of ADAMTS1 (AlphaFold ID AF-Q9UHI8-F1, visualized using PyMOL, version 2.5.4) and schematic of ADAMTS1 variants, showing the predicted molecular weight of zymogen and mature forms. Molecular masses were calculated based on ExPASy ProtParam tool. All constructs contained a C-terminal FLAG tag (not shown). *B* and *C*, expression of ADAMTS1 domain deletion variants. ADAMTS1 variants were transfected in HEK293T cells. Heparin was added 4 h post-transfection to release ECM/cell-bound forms. Conditioned media (*B*) and cell lysates (*C*) were analyzed by immunoblot under reducing conditions (5% β-mercaptoethanol) using anti-FLAG antibodies. Actin was used as a loading control. *Red stars* indicate the zymogens, and *blue stars* indicate cleavage products. *D* and *E*, purification of ADAMTS1 variants. Seventy-two hours post-transfection, CM was harvested by centrifugation and purified with anti-FLAG Sepharose. Purified samples were analyzed by (*D*) anti-FLAG immunoblot (50 ng/lane) and (*E*) CBB staining (2000 ng/lane) under reducing conditions (5% β-mercaptoethanol). Concentrations of ADAMTS1 were measured using optical absorbance. *Red stars* indicate the zymogens, *purple stars* indicate differentially *N*-linked glycosylated zymogen forms, *white stars* indicate differentially *N*-linked glycosylated mature forms, *blue stars* indicate cleavage products following Pro removal, and *black stars* indicate aggregated forms. CBB, Coomassie Brilliant Blue; CM, conditioned media; ECM, extracellular matrix; IB, immunoblot.

ADAMTS1 is a secreted multidomain protease. From the N-terminus, its domain organization consists of a prodomain (Pro), a catalytic metalloproteinase (Mp) domain, a disintegrin-like (Dis) domain, a thrombospondin type I (TSR) motif, a cysteine-rich (CysR) domain, a spacer (Sp) domain, and two additional C-terminal TSR motifs (Fig. 1*A*). To assess the contribution of individual domains to versican recognition and cleavage, we generated a series of variants where a domain was sequentially removed at the C-terminus (Fig. 1*A*). All constructs contained a C-terminal FLAG tag (DYKDDDDK) for detection and purification. Since we have previously shown that the C-terminal TSR motif is not involved in versicanase activity of ADAMTS5 (31), we hypothesized that ADAMTS1 had similar requirements. Therefore, the first ADAMTS1 variant, MDTCS, was truncated after the Sp. The other variants were deleted after the CysR (MDTC), central TSR motif (MDT), Dis (MD), or included the Mp domain alone (M). These constructs were transiently expressed in HEK293T cells in the presence and absence of heparin to release both ECM- and cell-bound ADAMTS1 (32). Both conditioned media (CM) (Fig. 1*B*) and cell lysates (Fig. 1*C*) were subjected to immunoblotting analysis using anti-FLAG antibodies for detection. All variants were expressed and secreted in the CM at different levels (Fig. 1*B*). Overall, the observed molecular weights matched well with the ones computed using the Expasy ProtParam tool (https://web.expasy.org/protparam/). A trend was observed with the shortest forms being secreted at the highest levels. In the CM, variants MD and M showed bands corresponding to the zymogen forms, most likely due to overwhelmed processing capacity at the highest expression levels (Fig. 1*B*). Bands corresponding to the zymogens and the mature forms were visible also in the cell lysates (Fig. 1*C*). Both WT ADAMTS1 and MDTCS showed C-terminal cleavage fragments in the CM (Fig. 1*B*), with cleavage products being more prominent in the cell lysate (Fig. 1*C*). The size of these species, matching that of the Sp and the two C-terminal TSR motifs, likely reflects autolytic/nonautolytic cleavages occurring N-terminal to or within the Sp (33, 34).

In the CM, WT ADAMTS1 and MDTCS could only be detected in the presence of heparin, while secretion of the variants lacking the Sp domain was not affected by the presence of heparin (Fig. 1*B*). This data suggested that the Sp is critical for the binding to the ECM/cell surface. In the lysate, where high levels of zymogen were present, zymogen forms of MD and M migrated as doublets representing differential *N*-linked glycosylated forms as observed before (35) (Fig. 1*C*).

Having confirmed that all the constructs were expressed, large scale transfections were performed to obtain the proteins necessary for functional studies. Based on the results from small scale transfections (Fig. 1*B*), heparin was added 4 h post-transfection only to the WT and MDTCS constructs. CM was purified with anti-FLAG affinity resin. Resin-bound ADAMTS1 was washed with 1 M NaCl to remove heparin and other contaminants (31, 32), before elution with FLAG peptides. Purified preparations were subsequently analyzed by immunoblot analysis using anti-FLAG antibodies for detection (Fig. 1*D*) and the purity was assessed by Coomassie Brilliant Blue (CBB) (Fig. 1*E*). All preparations contained some degree of the zymogen form as well as cleavage products. In the M preparations, some high molecular weight forms were present, under both reducing (Fig. 1*E*) and nonreducing conditions (Fig. S1), indicating that these are hydrophobic aggregates which are partially SDS-resistant, rather than disulfide aggregates.

### Peptidolytic activity of ADAMTS1 domain-deletion variants

**Figure 2:**
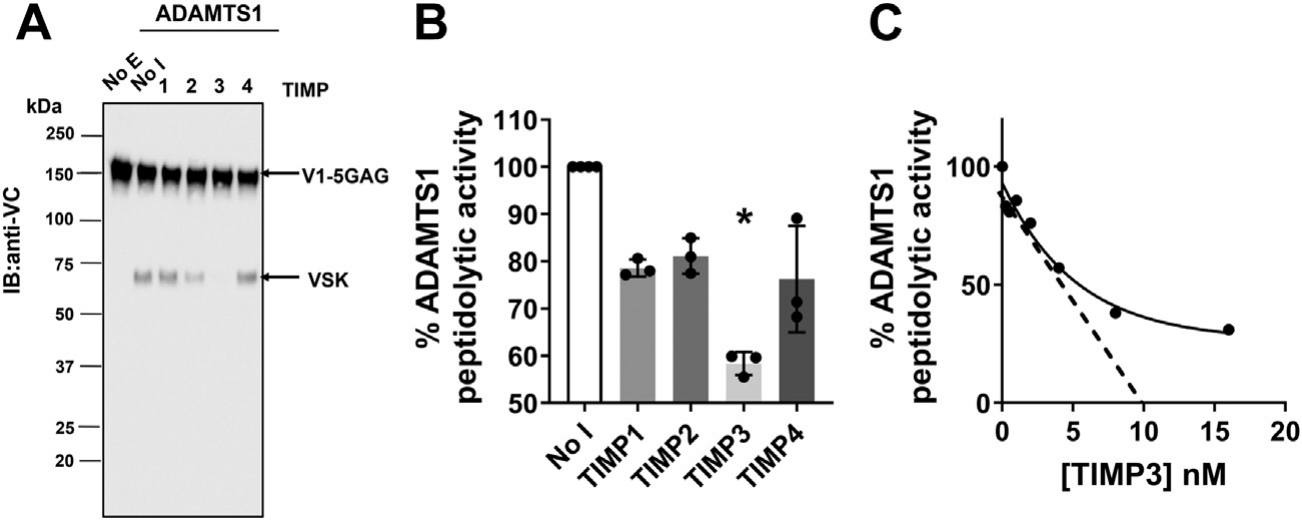
**Inhibition of ADAMTS1 by TIMPs.***A*, inhibition of versicanase activity by TIMP family members. TIMP1, TIMP2, TIMP3, and TIMP4 (each at 500 nM) were incubated with ADAMTS1 (100 nM) for 1 h at 37 °C before addition of V1-5GAG and digestion for 2 h. Following SDS-PAGE under reducing conditions (5% β-mercaptoethanol) and immunoblotting, FL V1-5GAG and versikine (VSK) were detected by the anti-Vc antibody. A representative immunoblot is shown (n = 2 independent experiments). *B*, inhibition of peptidolytic activity. TIMPs (each 25 nM) were incubated with a nominal concentration of 25 nM ADAMTS1 for 1 h at 37 °C before addition of the QF peptide substrate fluorescein-5(6)-carbonyl-Ala-Glu-Leu-Asn-Gly-Arg-Pro-Ile-Ser-Ile-Ala-Lys (5(6)-TAMRA) (3.5 μM) and digestion for 2 h. Following subtraction of the background (reactions not containing ADAMTS1), values were converted into percentage of ADAMTS1 activity in the absence of TIMPs and reported as average ± SD (n = 3, each point representing a technical replicate), *p* < 0.05 by Mann-Whitney test. *C*, titration of ADAMTS1 with TIMP3. TIMP3 (0–16 nM) was incubated with ADAMTS1 (20 nM nominal concentration) at 37 °C for 1 h, and residual activity against the QF peptide FAM-AELNGRPISIAK-Tamra (3.5 μM) was determined. A representative titration curve is shown, each point representing a mean of two technical replicates. Final concentration of ADAMTS1 following titration was 10 nM. FL, full-length; No E, no enzyme; No I, no inhibitor; QF, Quenched-Fluorescent; TIMP, tissue inhibitor of metalloproteinase.

**Figure 3:**
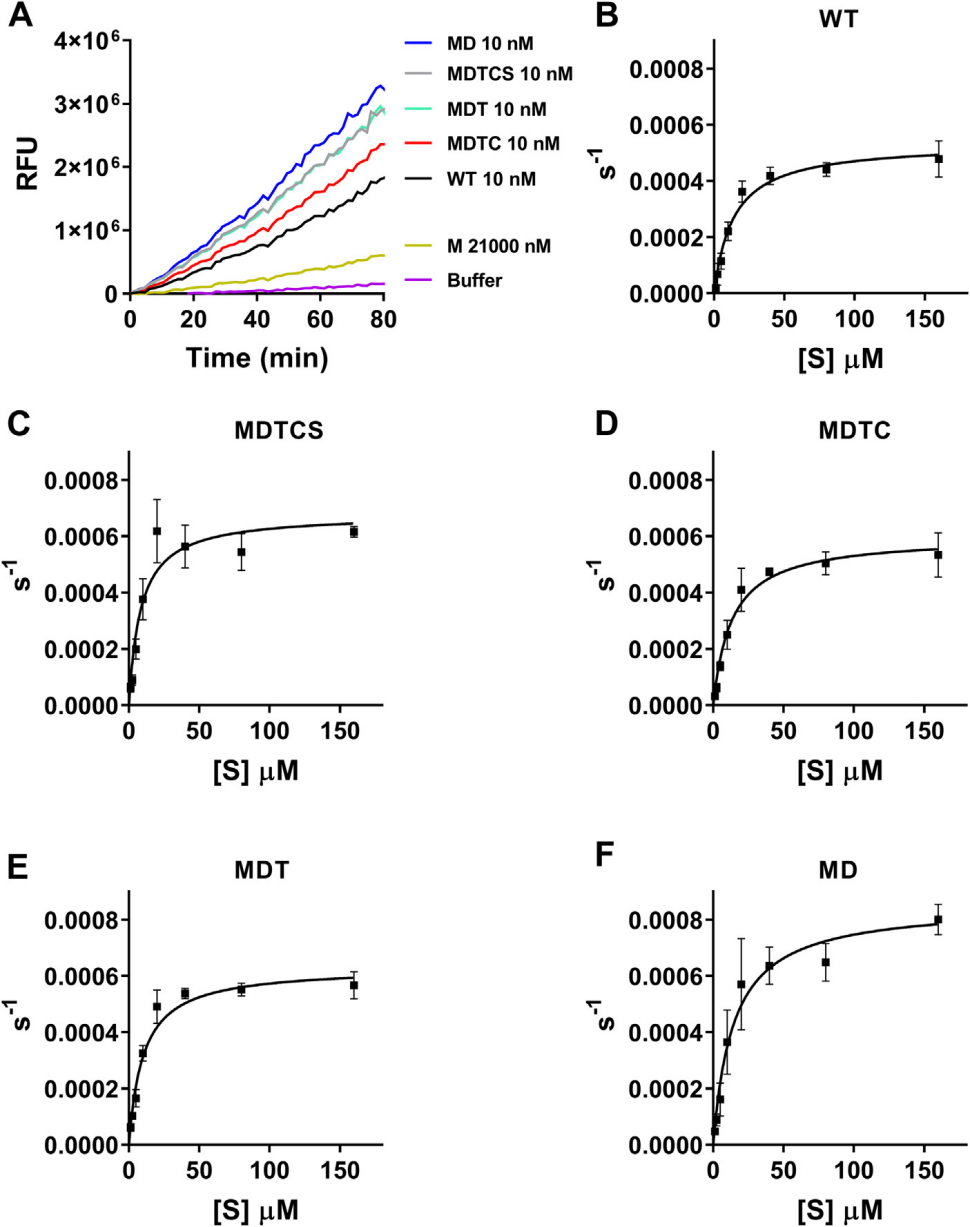
**Proteolytic activities of ADAMTS1 domain-deletion variants against a QF peptide substrate**. *A*, proteolytic activities of ADAMTS1 variants (10 nM) were tested using QF peptide fluorescein-5(6)-carbonyl-Ala-Glu-Leu-Asn-Gly-Arg-Pro-Ile-Ser-Ile-Ala-Lys (5(6)-TAMRA) (3.5 μM). Due to its low activity, variant M was tested at 21 μM. Fluorescent intensity was recorded with an excitation wavelength of 485 nm and an emission wavelength of 538 nm every min for 2 h, expressed as relative fluorescence units (RFU). Buffer indicates a reaction containing only buffer and substrate. A representative experiment is shown (n = 3 independent experiments). *B*–*F*, Michaelis–Menten curves for proteolysis of the QF peptide by ADAMTS1 variants. Data are plotted as turnover number *versus* substrate concentration and are presented as mean ± SD (n = 4 independent experiments). QF, Quenched-Fluorescent.

CBB and immunoblot analysis showed that ADAMTS1 preparations contained different levels of zymogen and mature forms, as well as degradation products (Fig. 1, *D* and *E*), which may lead to overestimation of their active concentrations if these were based on optical density. We hypothesized that, like ADAMTS4 and ADAMTS5 (31, 36, 37, 38), those ADAMTS1 variants containing at least the Mp and Dis domains would retain a fully functional active site. If so, ADAMTS1 concentrations could be determined using active-site titrations with tight-binding inhibitors (39), as routinely done for ADAMTS4, ADAMTS5 (31, 38), and ADAMTS7 (40). The four Tissue Inhibitors of Metalloproteinases (TIMPs) are endogenous ADAMTS inhibitors acting through the bidentate coordination of the active site zinc by the N-terminal α-amino group and the carbonyl group of a conserved N-terminal cysteine residue, thus binding in a 1:1 ratio (41). TIMP3 was reported as the most potent inhibitor of ADAMTS1 aggrecanase activity, followed by TIMP2, while TIMP1 and TIMP4 did not show significant inhibition (26). Initially, we confirmed these observations using V1-5GAG, a truncated versican variant (residues 21–694) (42), as a substrate (Fig. 2*A*). ADAMTS1 (100 nM) was incubated with different TIMPs (each at 500 nM) for 1 h before addition of V1-5GAG (100 nM) and incubation at 37 °C for 2 h. After deglycosylation, cleavage products were analyzed by SDS-PAGE and immunoblotting using the anti-Vc antibody, which recognizes an epitope spanning the Glu^441^-Ala^442^ cleavage site and thus detects both FL V1-5GAG and versikine (42). Under these conditions, TIMP3 showed complete inhibition of versikine generation, while TIMP2 showed only partial inhibition (Fig. 2*A*). Although this assay provided an indication that TIMP3 can potentially be used to titrate ADAMTS1 activity, it suffers from two major drawbacks: (1) it is based on a proteoglycan substrate which, due to its size, is known to engage distantly located exosites in the ancillary domains (31); (2) it provides only semiquantitative measurements, since it is based on immunoblot analysis. On the other hand, Quenched-Fluorescent (QF) peptide substrates are ideal for active-site titration since their size is restricted to the optimal distance between fluorophore and quencher to 4 to 17 residues (43). We found that ADAMTS1 cleaved the ADAMTS4 QF peptide substrate 5,6 fluorescein (FAM)-AELNGRPISIAK-carboxytetramethylrhodamine (TAMRA) at a minimal tested concentration of 25 nM (Fig. 2*B*). In agreement with the V1-5GAG data, TIMP3 was the most potent inhibitor of ADAMTS1 peptidolytic activity (Fig. 2*B*). Following on from our initial tests, we determined the concentrations of active ADAMTS1 by titration with increasing concentrations of TIMP3 using this QF substrate (Fig. 2*C*). Although TIMP3 did not achieve complete inhibition in this assay, the estimated concentrations were below the ones estimated by optical density, suggesting that TIMP3 can give a better estimate of active ADAMTS1 concentrations. TIMP3 was used then to titrate different batches of ADAMTS1 and its variants. Final yields of active ADAMTS1 were 115 mg/l for WT, 110 mg/l for MDTCS, 336 mg/l for MDTC, 130 mg/l for MDT, and 175 mg/l for MD. Titrated concentrations of WT ADAMTS1 and its variants generated similar cleavage curves for the QF peptide (Fig. 3*A*). However, variant M showed very modest peptidolytic activity, even at the highest concentration tested (21 μM) (Fig. 3*A*). As a consequence, concentration of variant M could not be reliably determined and was considered inactive.

We then determined the Michaelis–Menten constants for proteolysis of the QF substrate by ADAMTS1 variants (Fig. 3, *B*–*F*). All variants showed similar specificity constants (*k*_cat_/*K*_m_) compared to WT, while MDTCS showed a small but significant increase (Table 1). Taken together, these results suggest that ADAMTS1 domain-deletion variants retained a functional active site, with the exception of M that was excluded from further studies.

**Table 1:** Kinetic parameters for proteolysis of QF peptide substrate by ADAMTS1 variants

Variant	*K*_m_ μM (fold)	*k*_cat_ s^−1^ (fold)	*k*_cat_/*K*_m_^a^ 10^3^ M^−1^ s^−1^ (fold)
WT	13.5 ± 3.6 (1.0)	0.054 ± 0.004 (1.0)	4.2 ± 1.1 (1.0)
MDTCS	8.9 ± 1.3∗ (1.5)	0.067 ± 0.004∗ (0.8)	7.6 ± 1.3∗ (0.6)
MDTC	13.3 ± 1.5 (1.0)	0.060 ± 0.005 (0.9)	4.5 ± 0.71 (0.9)
MDT	10.3 ± 1.3 (1.3)	0.063∗ ± 0.003 (0.9)	6.2 ± 0.63 (0.7)
MD	15.8 ± 5.6 (0.9)	0.088∗ ± 0.009 (0.6)	6.1 ± 2.3 (0.7)

Results are expressed as mean ± SD (n = 4 independent experiments). ∗*p* < 0.05 compared to WT ADAMTS1 using two-tailed Mann-Whitney test. Fold changes were calculated as the ratio of the kinetic parameters of each variant *versus* WT.

### Proteoglycanase activity of ADAMTS1 domain-deletion variants

**Figure 4:**
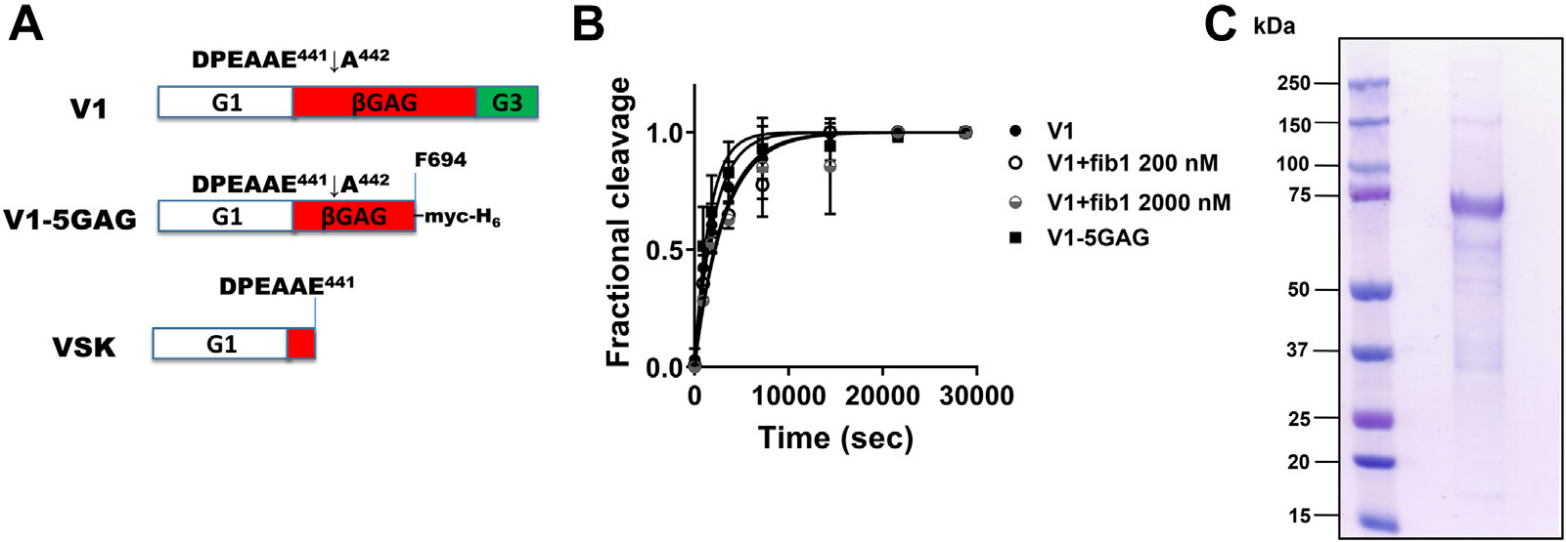
**Versicanase activity of ADAMTS1**. *A*, domain structure of FL V1, V1-5GAG, and versikine (VSK). *B*, time course experiments for the cleavage of V1-5GAG (50 nM) or V1 (50 nM) by ADAMTS1 (140 nM) in the presence and absence of fibulin1 (200 and 2000 nM). At the indicated timepoints, an aliquot was taken, stopped with EDTA, and the concentration of cleavage products was quantified by ELISA. Versikine was captured by anti-DPEAAE antibodies and detected by an anti-G1 antibody. Fractional cleavage was plotted against time. Data are present as mean ± SD (n = 3-6 independent experiments). The *solid lines* represent a nonlinear regression fit of the data as described in the Experimental procedures. *C*, CBB staining under reducing conditions (5% β-mercaptoethanol) of purified human recombinant fibulin1. CBB, Coomassie Brilliant Blue; Fib1, fibulin1; FL, full-length.

We have previously reported that ADAMTS1 versicanase activity is much weaker than that of related proteases ADAMTS4 and ADAMTS5; however, no quantitative measurements were reported (31). To determine kinetic parameters for ADAMTS1 versicanase activity, WT ADAMTS1 (140 nM) was incubated with either FL versican V1 or V1-5GAG (42) (Fig. 4*A*), and the samples were analyzed using our versikine ELISA where versikine is captured by a neoepitope anti-DPEAAE antibody and detected with an anti-G1 antibody (31). ADAMTS1 cleaved the two substrates with very similar specificity constants (*k*_cat_/*K*_m_), in the range of 3 × 10^3^ M^−1^ s^−1^ (Fig. 4*B* and Table 2). This data suggested that V1-5GAG contains all of the necessary binding sites for optimal cleavage at the Glu^441^-Ala^442^ site, as previously shown for ADAMTS4 and ADAMTS5 (31). Fibulin1, a member of the fibulin family of ECM proteins, has been proposed as a cofactor for ADAMTS1 aggrecanase (44) and versicanase activity (12). We therefore aimed to test if fibulin1 affected ADAMTS1 versicanase activity. Fibulin1 was expressed in HEK293T cells and purified using Ni-chromatography (Fig. 4*C*). WT ADAMTS1 enzymes showed very similar specificity constants in the presence and absence of 200 and 2000 nM fibulin1 (Fig. 4*B* and Table 2), suggesting that fibulin1 does not act as a cofactor for ADAMTS1 under these conditions.

**Table 2:** Kinetic parameters for proteolysis of V1 and V1-5GAG by ADAMTS1

Substrate	Cofactor	*k*_cat_/*K*_m_^a^ 10^3^ M^−1^ s^−1^	Fold reduction
V1	none	3.4 ± 1.1	−
V1	Fibulin1 200 nM	2.4 ± 0.46	1.38
V1	Fibulin1 2000 nM	2.22 ± 0.58	1.51
V1- 5GAG	none	4.2 ± 2.3	0.80

Values were determined by time course experiments at 50 nM substrate concentration and expressed as mean ± SD (n = 3–6 independent experiments). Fold reduction was calculated relative to the value obtained for V1 in the absence of cofactors.

**Figure 5:**
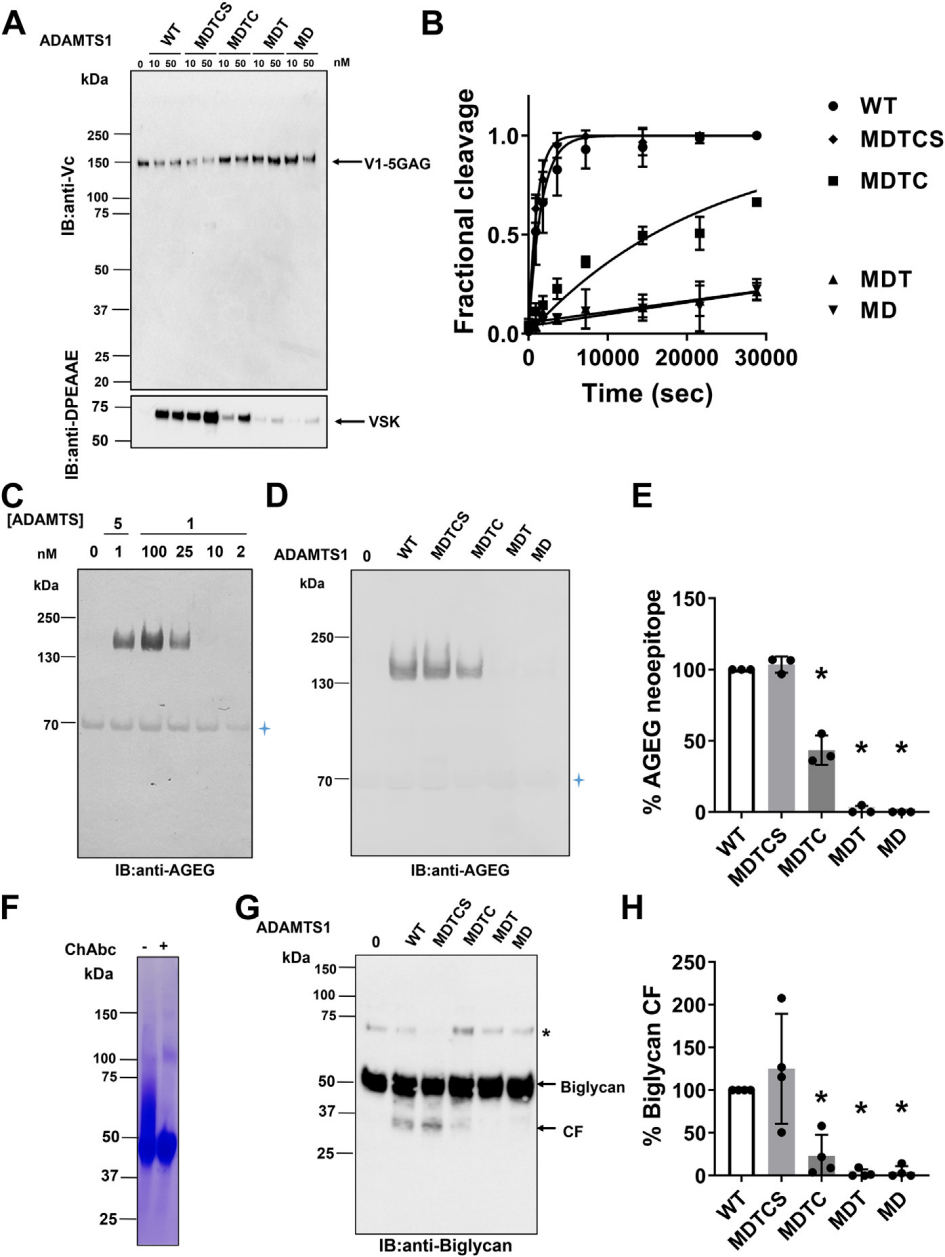
**Proteoglycanase activity of ADAMTS1 domain deletion variants.***A*, V1-5GAG (100 nM) was incubated with ADAMTS1 domain-deletion variants (10 nM and 50 nM) for 2 h at 37 °C before addition of EDTA. Samples were then deglycosylated, subjected to SDS-PAGE, and blotted either with the anti-Vc or anti-DPEAAE antibodies to detect cleavage fragments. *B*, time course experiments for cleavage of V1-5GAG (50 nM) by ADAMTS1 variants (140 nM). At the indicated timepoints, an aliquot of the reaction was taken, stopped with EDTA, and cleavage products were measured by ELISA. Fractional cleavage was plotted against time. The *solid lines* represent a nonlinear regression fit of the data as described in the Experimental procedures. Data are expressed as mean ± SD (n = 6 independent experiments). *C*, aggrecanase activity of ADAMTS1 compared to ADAMTS5. Bovine aggrecan (330 nM) was incubated with different concentrations of ADAMTS1 and ADAMTS5 (1 h, 37 °C). Samples were deglycosylated, subjected to SDS-PAGE, and detected using anti-AGEG neoepitope antibody, which specifically detects cleavage at the Glu^1953^-Ala^1954^ bond. *Blue asterisk* indicates aspecific bands. Representative immunoblot of two independent replicates. *D*, aggrecanase activity of ADAMTS1 domain deletion variants. Representative immunoblot of three independent replicates. *Blue asterisk* indicates aspecific bands. *E*, intensities of the bands corresponding to anti-AGEG reactive cleavage fragments were quantified by using Image Lab 6.1. Results were expressed as mean ± SD (n = 3 independent experiments). ∗*p* < 0.05. *F*, CBB staining of purified biglycan under reducing conditions. The presence of chondroitin-sulfate chains was confirmed by the shift in mobility upon chondroitinase ABC (ChAbc) treatment. *G*, Biglycan (100 nM) was incubated with ADAMTS1 domain-deletion variants (50 nM) for 24 h at 37 °C. Samples were then deglycosylated, subjected to SDS-PAGE, and blotted with a polyclonal anti-biglycan antibody to detect both FL biglycan and cleavage fragments (CFs). Note the absence of cleavage fragments in the buffer control (0). The asterisk indicates a biglycan-specific band detected in previous studies (36). *H*, intensities of cleavage fragment bands were quantified by using Image Lab 6.1. Results were expressed as mean ± SD (n = 4 independent experiments). All gels were run under reducing conditions (5% β-mercaptoethanol). ∗*p* < 0.05. IB, immunoblot. VSK, versikine; CBB, Coomassie Brilliant Blue; FL, full-length.

Since WT ADAMTS1 cleaved FL V1 and V1-5GAG with similar efficiency (Table 2), the latter was used to compare the versicanase activity of the different variants. Initially, for a qualitative comparison, purified V1-5GAG (100 nM) was incubated with ADAMTS1 (10 and 50 nM) for 2 h at 37 °C, the reactions were stopped with EDTA, deglycosylated, and analyzed by SDS-PAGE followed by immunoblotting (Fig. 5*A*). In addition to anti-Vc, the neoepitope antibody anti-DPEAAE, which detects versikine fragments, was used to visualize versican cleavage. WT ADAMTS1 converted most of the V1-5GAG substrate into versikine at a minimal concentration of 10 nM. Removal of the two C-terminal TSRs moderately increased versicanase activity. However, this was markedly reduced following the removal of Sp (variant MDTC), resulting in only a minor proportion of the substrate being converted into versikine at 10 nM. Further reduction was observed following the removal of CysR (variant MDT) and the central TSR (MD).

Versicanase activity of the ADAMTS1 variants was subsequently tested in time-course experiments followed by versikine-ELISA (Fig. 5*B* and Table 3). Removal of the C-terminal TSRs (MDTCS) resulted in a modest increase in versicanase activity, whereas deletion of Sp (MDTC) reduced versicanase activity by 16-fold. Removal of CysR (MDT) led to a dramatic, 64-fold reduction, while further removal of the central TSR (MD) did not result in any further decrease in versicanase activity. This data suggested that the Sp and CysR ancillary domains are essential for ADAMTS1 versicanase activity.

**Table 3:** Kinetic parameters for proteolysis of V1-5GAG by ADAMTS1 domain deletion variants

Variant	*k*_cat_/*K*_m_^a^ 10^3^ M^−1^ s^−1^	Fold reduction
WT	4.2 ± 2.3	−
MDTCS	7.3 ± 2.2∗	0.58
MDTC	0.26 ± 0.057∗∗	16.2
MDT	0.066 ± 0.013∗∗	63.6
MD	0.061 ± 0.028∗∗	68.9

Values determined by time course experiments at 50 nM substrate concentration. Results given in nanomolar and expressed as mean ± SD (n = 6 independent experiments). ∗*p* < 0.05, ∗∗*p* < 0.01, compared to WT ADAMTS1 using two-tailed Mann-Whitney test. Fold reduction was calculated relative to the value obtained in the presence of WT ADAMTS1.

We then aimed to extend these findings to other proteoglycan substrates. Since we confirmed that ADAMTS1 did not exhibit evident aggrecanase activity at the canonical site Glu^392^-Ala^393^ (Fig. S2), we tested if it could cleave aggrecan at the Glu^1953^-Ala^1954^ bond (Glu^1790^-Ala^1791^ in bovine aggrecan, UniProt ID P13608-1), a site that is favored by both ADAMTS4 and ADAMTS5 (45). Using a specific neoepitope antibody (46), we detected robust aggrecanase activity at this site, although this was approximately 100-fold lower than ADAMTS5 (Fig. 5*C*). We then proceeded to investigate the ability of ADAMTS variants to cleave at the Glu^1953^-Ala^1954^ bond (Fig. 5, *D* and *E*). While variant MDTCS cleaved aggrecan as efficiently as the WT, aggrecanase activity of MDTC was severely decreased, suggesting that the Sp is important for recognition and cleavage of aggrecan. Further removal of the CysR (variant MDT) abolished the aggrecanase activity completely.

Having recently identified the small leucine-rich proteoglycan biglycan as an ADAMTS1 substrate (21), we proceeded to investigate how removal of individual domains affects ADAMTS1 biglycanase activity. A construct encoding for FL biglycan with a 6x-His C-terminal tag was transiently transfected in HEK293T cells and purified using Ni-chromatography. Treatment with chondroitinase ABC resulted in decreased polydispersity of the purified preparation during migration on SDS-PAGE (Fig. 5*F*), thus confirming that recombinant biglycan contains chondroitin sulfate chains. ADAMTS1 variants (50 nM) were incubated with biglycan (1130 nM) for 24 h at 37 °C. Digested samples were deglycosylated with chondroitinase ABC and analyzed by SDS-PAGE and immunoblotting with a polyclonal anti-biglycan antibody (Fig. 5*G*). As observed before (21), WT ADAMTS1 biglycanase activity generated a ∼30 kDa fragment which could be quantified by densitometric analysis (Fig. 5*H*). Removal of the two C-terminal TSR motifs (variant MDTCS) had no effect on biglycanase activity, whereas deletion of the Sp (MDTC) reduced generation of the 30 kDa biglycan fragment by 75%. Further removal of the CysR (MDT) completely abolished ADAMTS1 biglycanase activity.

Overall, these results indicate a similar recognition and binding pattern for all three proteoglycans, with major exosites being present in the Sp and CysR domains.

### Identification of exosites in the Sp domain

**Figure 6:**
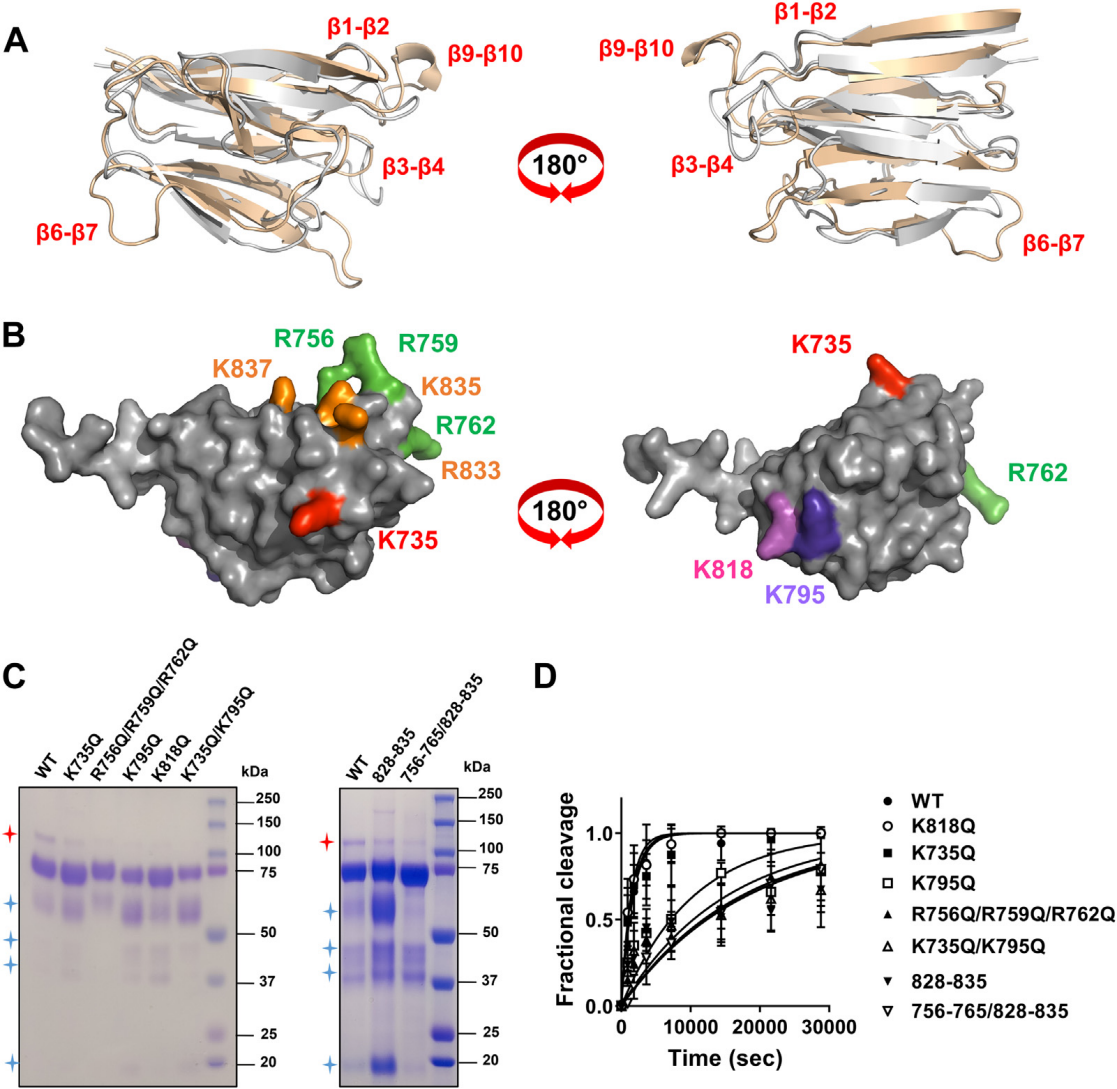
**Identification of exosites in ADAMTS1 spacer domain.***A*, superimposition of the AlphaFold model of ADAMTS1 Sp domain (AlphaFold ID:AF-Q9UHI8-F1) (*gray*) and crystal structure of ADAMTS13 (pdb 3GHN) (*light orange*). *B*, surface representation of the predicted ADAMTS1 Sp domain (AlphaFold ID:AF- AF-Q9UHI8-F1) highlighting the positively charged residues mutated to glutamine. Models were visualized using PyMOL. *C*, CBB staining of purified ADAMTS1 Sp variants under reducing conditions (5% β-mercaptoethanol). *D*, time course experiments for the cleavage of V1-5GAG (50 nM) by ADAMTS1 variants (140 nM). At the indicated time points, an aliquot of the reaction was taken, stopped with EDTA, and cleavage products were measured by ELISA. Fractional cleavage was plotted against time. The *solid lines* represent a nonlinear regression fit of the data as described in the Experimental procedures. Data are expressed as mean ± SD (n = 3–6 independent experiments). CBB, Coomassie Brilliant Blue.

**Figure 7:**
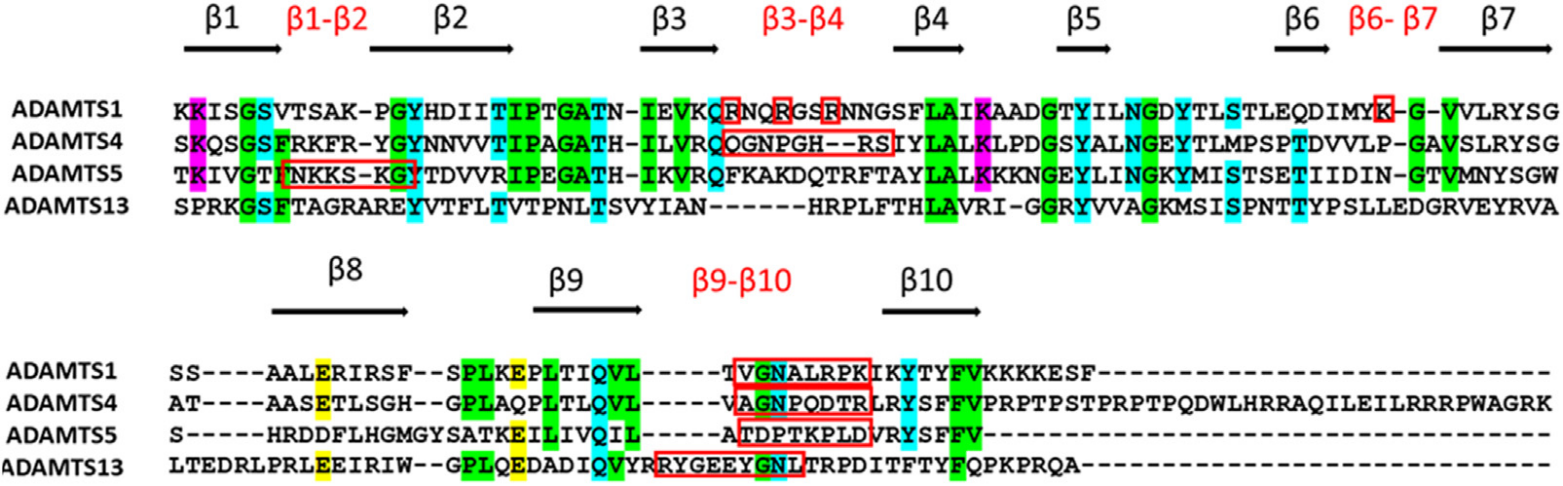
**Sequence alignment of the Sp domain of human ADAMTS1, ADAMTS4, ADAMTS5, and ADAMTS13**. Alignment was performed in Clustal Omega (https://www.ebi.ac.uk/Tools/msa/clustalo/) and visualized using MView (https://www.ebi.ac.uk/Tools/msa/mview/). UniProt accession numbers were Q9UHI8 (ADAMTS1, aa 725–749), O75173 (ADAMTS4, aa 686–837), Q9UNA0 (ADAMTS5, aa 732–874), and Q76LX8 (ADAMTS13, aa 556–685). Percentage identities were 23.9, 17.6, and 14.2%, respectively, compared with ADAMTS13. Beta strands and interconnecting loops are indicated. Exosite residues are highlighted by *red rectangles*. Amino acids conserved in at least three of the four enzymes are colored according to physicochemical properties (*purple*, positively charged; *yellow*, negatively charged; *green*, apolar; *cyan*, polar).

Having established that the Sp is a major determinant of ADAMTS1 proteoglycanase activity, we aimed to identify the residues involved in the interaction between Sp and proteoglycans, the so called exosites. So far, the structure of ADAMTS1 Sp has not been resolved. Four ADAMTS1 structures have been deposited in the Protein Data Bank (https://www.ebi.ac.uk/pdbe/), only covering the Mp and Dis domains. In ADAMTS13, the only ADAMTS family member for which the 3D structure of the ancillary domains has been experimentally resolved, the Sp consists of 10 β-strands in a jelly-roll topology (47, 48). The fact that this is the case also for ADAMTS1 is supported by the high conservation between the sequences of the ADAMTS1 and ADAMTS13 Sp (24.82% identity; 40.15% similarity as computed using the Sequence Manipulation Suite, https://www.bioinformatics.org/sms2/ident_sim.html) as well as the prediction of AlphaFold (version 1 July 2021) (49, 50) (Fig. 6*A*). While most conserved residues lie in the beta strands, the sequence and orientation of the connecting loops is highly divergent (Figs. 6*A* and 7), suggesting that these regions are the determinants of substrate specificity. We have previously reported that in the case of closely related ADAMTS4 and ADAMTS5 exosites lie in at least two of these loops (31), one of their common features being the presence of positively charged residues. To identify ADAMTS1 exosites, we performed a glutamine scanning mutagenesis where lysine/arginine residues in the Sp loops were mutated into glutamine (Fig. 6*B*). Six variants were generated and transiently transfected in HEK293T (alongside WT ADAMTS1, here chosen as a positive control for expression) in the presence and absence of heparin: K735Q (β1-β2 loop), R756Q/R759Q/R762Q (β3-β4), K795Q (β6-β7), K735Q/K795Q (β1-β2 and β6-β7), K818Q (β8-β9), and R833Q/K835Q/K837Q (β9-β10). Five of these were found to be secreted in the CM (Fig. S3*A*): K735Q, R756Q/R759Q/R762Q, K795Q, K735Q/K795Q, and K818Q. Like WT ADAMTS1, levels of these five variants in the CM increased in the presence of heparin, suggesting that mutations at these residues did not disrupt a heparin-binding site. Only one variant, R833Q/K835Q/K837Q in the β9-β10 loop, was not secreted in the CM. We hypothesized that simultaneous mutation of these three charged residues had a detrimental effect on the folding/stability of the protein, since we were not able to detect the protein even in the cell lysate (Fig. S3*B*). To circumvent this, we decided to replace the entire ADAMTS1 loop with homolog residues in another ADAMTS family member, since we have previously shown that such ADAMTS chimeras express well (31). In this case, we adopted a conservative approach where the least divergent loop, that of ADAMTS4 (Fig. 7), was selected for this substitution. Since ADAMTS1, ADAMTS4, and ADAMTS5 present a positively charged residue in the position corresponding to K837 (Fig. 7), it is feasible that this also plays a structural role. Taking into account these structural indications, we generated an additional variant, 828-835, where a portion of the β9-β10 loop covering residues 828-835 (^828^VGNALRPK^835^, positively charged residues in bold) was substituted with that of ADAMTS4 (^788^AGNPQDTR^795^). This variant was successfully expressed and purified, just like the other five Sp variants (Fig. 6*C*). Following determination of their concentrations by active site titrations, all six variants were tested in the versicanase assay (Fig. 6*D* and Table 4). Variants K735Q and K818Q, bearing mutations in the β1-β2 and β8-β9 loops, respectively, did not show any significant decrease in versicanase activity, suggesting that these residues are not involved in versican recognition. On the other hand, the triple variant R756Q/R759Q/R762Q, bearing mutations in the β3-β4 loop, showed a ∼5-fold decrease in versicanase activity. Variant K795Q showed a ∼6-fold decrease in versicanase activity, similar to K735Q/K795Q. Finally, variant 828-835 showed a ∼10-fold decrease in versicanase activity, a degree of reduction close to the one observed upon deletion of the entire Sp (variant MDTC, Table 3). We then aimed to assess if by combining mutations in the β3-β4 and β9-β10 loops, which compose a single interface (Fig. 6*A*), we would be able to observe a further reduction in versicanase activity. Because addition of multiple single point mutations was likely to affect secretion/expression of the resulting variant, we decided to substitute residues in the β3-β4 loop (^756^RNQRGSRNNG^765^) with those of ADAMTS4 (^717^QGNPGHRS^724^) in variant 828-835. The resulting variant, 756-765/828-835, was indeed successfully expressed and purified (Fig. 6*C*) and its versicanase activity found to be reduced ∼9-fold compared to WT (Table 4), suggesting that ADAMTS1 can alternatively use the β3-β4 or β9-β10 loop to recognize versican.

**Table 4:** Kinetic parameters for proteolysis of V1-5GAG by ADAMTS1 Sp variants

Variant	Loop mutations	*k*_cat_/*K*_m_^a^ 10^3^ M^−1^ s^−1^	Fold reduction
WT	−	4.2 ± 2.3	−
K735Q	β1-β2	4.8 ± 2.6	0.88
R756Q/R759Q/R762Q	β3-β4	0.87 ± 0.66∗∗	4.8
K795Q	β6-β7	0.73 ± 0.59∗∗	5.8
K735Q/K795Q	β1-β2; β6-β7	0.57 ± 0.40∗∗	7.4
K818Q	β8-β9	6.05 ± 4.00	0.69
R833Q/K835Q/K837Q	β9-β10	ND	ND
828-835	β9-β10	0.41 ± 0.07∗∗	10.2
756-765/828-835	β3-β4; β9-β10	0.46 ± 0.23∗∗	9.1

Values were determined by time course experiments at 50 nM substrate concentration and expressed as mean ± SD (n = 3-6 independent experiments). ∗∗*p* < 0.01, compared to WT ADAMTS1 using two-tailed Mann-Whitney test. Fold reduction was calculated relative to the value obtained for in the presence of WT ADAMTS1.

Overall, the combined results from the glutamine scanning mutagenesis and loop substitution studies suggest that residues R756Q/R759Q/R762Q in the β3-β4 loop, K795Q in the β6-β7 loop, and 828-835 in the β9-β10 loop comprise exosites involved in ADAMTS1 versicanase activity.

## Discussion

Notwithstanding the essential role exerted by ADAMTS1 versicanase activity in processes such as cardiac and urogenital development (4, 14), ovulation (51), neoangiogenesis, and cell invasion (33, 52), its underlying molecular mechanisms have remained elusive. A reason for this has been the difficulty in reconciliating the *in vivo* relevance of ADAMTS1 with data showing poor activity in pure component systems (24, 31). The lack of quantitative analysis further hampered the elucidation of the ADAMTS1–versican interactions. Here, we provided the first quantitative determination of ADAMTS1 versicanase activity. We showed that ADAMTS1 cleaves FL versican with a *k*_cat_/*K*_m_ value of 3.6 × 10^3^ M^−1^ s^−1^, a rate that is approximately 1000-fold lower than that for ADAMTS5 and 50-fold lower than ADAMTS4 (31). Due to this weak versicanase activity, to achieve complete proteolysis of the substrate within a reasonable amount of time, we had to use a 2.8 enzyme:substrate ratio. Under these conditions, it is possible that ADAMTS1 may cleave with different efficiencies at alternative sites, thus resulting in a lower apparent *k*_cat_/*K*_m_ value. Using mass spectrometry, we have previously identified additional cleavage sites in V1 by ADAMTS1 at Glu^768^-Leu^769^, Gln^1027^-Leu^1028^, and Glu^923^-Arg^924^ (30). However, only cleavage sites upstream of the ‘canonical’ versicanase site (Glu^441^-Ala^442^) will result in a decreased signal in our versikine ELISA, since this relies on an anti-G1 domain for detection (31). It is therefore unlikely that we have underestimated ADAMTS1 versicanase activity due to overdigestion. The very similar specificity constants obtained for FL V1 and V1-5GAG, which is truncated after Phe^694^, support the notion that additional cleavage events upstream of the Glu^441^-Ala^442^ bond do not significantly affect ADAMTS1 cleavage at this site since we demonstrated that ADAMTS1 cleaves V1-5GAG exclusively at the ‘canonical site’ (30). The lack of available assays to measure peptides generated at the three downstream cleavage sites in V1 FL makes it very challenging to establish which is the favored proteolytic event. A comparison of the relative abundance of semitryptic peptides suggested that cleavages at Glu^768^-Leu^769^, Gln^1027^-Leu^1028^, and Glu^923^-Arg^924^ are as favored as that at the ‘canonical’ site Glu^441^-Ala^442^ (30).

Another factor that can potentially affect the measured catalytic efficiency of ADAMTS1 is the presence of truncated species in our WT enzyme preparation. Such forms, which were observed in previous studies (33, 34), may lack one or more exosites in the Sp and CysR domains, while the isolated fragments may potentially compete with the FL enzyme for versican binding. Unfortunately, their removal, for example by size-exclusion chromatography, is unfeasible due to the low yields of FL ADAMTS1 and the possibility of further autolytic processing during additional purification steps. C-terminal processing is a characteristic feature of ADAMTS proteoglycanases such as ADAMTS5, affecting both recombinant and endogenous expression (53). It is therefore likely that the relative order of versicanase activity is maintained, given that the vast majority of the enzyme preparation is in its mature, unprocessed form.

Interestingly, there are indications that the ADAMTS1 has an intrinsically lower proteolytic activity than other proteoglycanases, such as ADAMTS4. The *k*_cat_ value of ADAMTS1 for the QF substrate fluorescein-5(6)-carbonyl-Ala-Glu-Leu-Asn-Gly-Arg-Pro-Ile-Ser-Ile-Ala-Lys-(5(6)-TAMRA) (Table 1), known to engage only the Mp domain, is >20-fold lower than that of ADAMTS4 (54). This observation suggests that ADAMTSs achieve a higher proteoglycanase activity by a combination of high-affinity exosites and highly efficient catalytic machineries, as previously shown by interchanging ADAMTS4 and ADAMTS5 ancillary domains (37).

To reconciliate the remarkable cardiovascular role of ADAMTS1 with its poor versicanase activity, cofactors such as fibulin1 have been proposed (55). Fibulins are functional components of the basement membrane and elastic fibers (56). Fibulin1 is expressed in the developing vessels where it regulates migration of smooth muscle cells and endothelial cells (57, 58, 59). Four isoforms (A-D) have been reported, arising by differential splicing of the C-terminal globular domain III (60). Fibulin1 binds to various ECM proteins including nidogen1, fibronectin, and aggrecan (56, 61). Isoforms C and D also bind to the versican G3 domain with relatively high affinity (*K*_d_ value: 14–38 nM) through their epidermal growth factor–like repeats in domain II (62). A yeast two-hybrid screen and coimmunoprecipitation studies have shown that fibulin1 binds to the two C-terminal ADAMTS1 TSR motifs through its three C-terminal EGF-like repeats, a region that is common to both isoforms C and D (44). Using SDS-PAGE followed by immunoblot, Lee *et al.* have shown that this interaction enhanced ADAMTS1 aggrecanase activity (44). In contrast, using quantitative enzyme kinetics, we demonstrated that fibulin1 was not able to enhance ADAMTS1 versicanase activity. Either ADAMTS1 adopts different conformations upon binding the two large aggregating proteoglycans, thus making fibulin1 an inefficient cofactor for versicanase activity, or under our experimental conditions (up to 20-fold excess over ADAMTS1, 2 μM, *i.e.*, above the reported *K*_D_ value of 1 μM (44)), fibulin1 was not able to enhance ADAMTS1 proteoglycanase activity.

The reasons for the discrepancy between *Adamts1* mouse models (14) and its poor proteoglycanase activity *in vitro* remains to be elucidated. It is possible that there are intrinsic differences between the mouse and human orthologs. However, the two orthologs exhibit a high percentage amino acid identity across all domains (82.6%). In particular, the Sp is well conserved (85.60% amino acid identity), with minimal differences in the exosite-containing loops (Fig. S4). Such a high sequence conservation suggests similar substrate preferences and specific activities, but this conclusion must be substantiated with experimental *in vitro* data on mouse ADAMTS1 versicanase activity. Other mechanisms that may explain the knockout phenotypes may be a compensative effect where low intrinsic ADAMTS1 activity is balanced by high expression levels or an indirect effect on versicanase activity mediated by ADAMTS1. While *Adamts1* KO mice manifested a range of phenotypes characterized by accumulation of versican (9), genetic ablation of more potent versicanases such as ADAMTS4 and ADAMTS5 resulted in much milder phenotypes (63, 64). Such a striking difference strongly indicates that ADAMTS1 is essential to maintain adequate levels of versican during the development, in tissues where the expression of both ADAMTS4 and ADAMTS5 is limited.

To characterize the structural determinants of ADAMTS1 proteoglycanase activity, five C-terminal domain deletion variants were expressed and purified. The Sp domain was required for ECM/cell binding, as ADAMTS1 variants devoid of the Sp were released into the medium even in the absence of heparin. This well agrees with early observations showing that the region C-terminal to the Dis was involved in ECM-binding (32) and that matrix metalloproteinase-dependent cleavage within the Sp led to release of the mature protein into the medium (34). Although the Sp does not contain a consensus sequence for heparin binding (XBBXBX, where B indicates a basic residue and X any other amino acid) (65), it presents several positively charged residues that can potentially interact with negatively charged GAGs in the ECM (Fig. 7). Although we were able to detect the mature, activated form of ADAMTS1 in the cell lysate, the secreted species were predominantly activated, suggesting that Pro removal mainly occurred in the late trans-Golgi pathway or extracellularly. The fact that this proteolytic event is mediated by furin, a ubiquitously expressed serine protease, was shown by Rodrigruez-Manzaneque *et al.* using a specific furin inhibitor (34). Other than Pro removal and ECM-binding, an additional mechanism to regulate ADAMTS1 levels is represented by receptor-dependent endocytosis. We have recently shown that ADAMTS1 binds to Low Density Lipoprotein receptor protein 1 (LRP1) (66), thus joining ADAMTS4 (67) and ADAMTS5 (68) among the known LRP1 ligands.

In contrast to the other deletion variants, ADAMTS1 M (consisting only of the Mp) was not proteolytically competent. The crystal structure of the ADAMTS1 MD (UniProt ID 2JIH) shows that the Dis domain packs closely against the Mp, indicating that the two domains are structurally integrated (Fig. S5*A*) (69), similar to what occurs in ADAMTS4 and ADAMTS5 (70), where the isolated Mp domain is proteolytically inactive (36). The Mp domain shows the presence of several exposed hydrophobic residues on the Mp/Dis interface (Fig. S5*B*), which may explain the presence of aggregated species observed on SDS-PAGE under both reducing and nonreducing conditions (Figs. 1*E* and S1). With the exception of ADAMTS1 M, all the tested variants showed similar specificity constants against the QF peptide, suggesting that this did not engage residues located in the ancillary domains. The QF peptide was thus the ideal substrate to determine enzyme concentration by active site titrations as shown before for other ADAMTS family members (31, 54, 71).

Using both versican and the QF peptide as substrates, we quantitatively demonstrated that among the four TIMPs, TIMP3 is the most potent ADAMTS1 inhibitor, as previously shown using aggrecan (26). Deletion of the two C-terminal TSR motifs (variant MDTCS) modestly increased ADAMTS1 versicanase activity. The AlphaFold model suggests that the C-terminal TSRs may restrain access to the upstream ancillary domains, in particular the Sp and CysR (Fig. 1*A*). Although these predictions must be evaluated critically in the absence of an experimentally resolved structure, they raise the intriguing possibility of a partially autoinhibitory function for the C terminal TSRs. In comparison, deletion of the single C-terminal TSR did not affect versicanase activity of ADAMTS5 (31). Like ADAMTS5 (31), in ADAMTS1, both the Sp and CysR were necessary for full versicanase activity, while in ADAMTS4, deletion of the Sp was sufficient to almost abolish versicanase activity.

We have previously shown that ADAMTS4 and ADAMTS5 engage their proteoglycan substrates using the same ancillary domains and residues (exosites) (31). Here, we showed that this is the case also for ADAMTS1 where the Sp and CysR were essential also for aggrecan and biglycan cleavage. Kuno *et al.* (23) have also reported that the Sp and CysR are essential for aggrecanase activity, while the two C-terminal TSRs were not involved. Biological processes that affect domain composition by removing one or more ancillary domains, such as alternative splicing or C-terminal processing, may therefore impact the ability of ADAMTS1 to recognize and cleave proteoglycans.

In this and in a previous study (21), we failed to detect consistent cleavage of bovine aggrecan by ADAMTS1 at the canonical site Glu^392^-Ala^393^, in a range of concentrations (0.1–500 nM), where the Iruela-Arispe group detected the presence of NITEGE neoepitope present on the C-terminal aggrecan fragment (26). The reasons for this discrepancy are at present unclear, although species differences in the substrate (bovine *versus* rat aggrecan) and/or in enzyme preparations may be possible explanations. Notwithstanding these differences, we can conclude that ADAMTS1 is a much weaker aggrecanase than ADAMTS4 and ADAMTS5, which cleave aggrecan at the Glu^392^-Ala^393^ and Glu^1953^-Ala^1954^ bonds in the low nanomolar range (45).

Biglycan belongs to the small leucine-rich proteoglycan family, characterized by the presence of 12 leucine-rich repeats and one N-terminal cysteine-rich cluster (72), and plays a role in the assembly of collagen fibrils (73). Among the ADAMTS family members, ADAMTS4 and ADAMTS5 also share the ability to cleave biglycan, while ADAMTS8 lacks biglycanase activity (21).

We hypothesized that, in the ADAMTS family, substrate specificity is dictated by ‘hypervariable’ loops engrafted within the conserved beta-strand residues of the Sp domain (Fig. 7) (31). ADAMTS4 employs loops β3-β4 (aa 717–724) and β9-β10 (aa 788–795), while ADAMTS5 contacts proteoglycans through loops β1-β2 (aa 739–744) and β9-β10 (aa 837–844) (31). The β9-β10 loop is also used by ADAMTS13, which lacks proteoglycanase activity, to bind its physiological substrate, von Willebrand Factor (74). Since ADAMTS1 exosites also lie in the β9-β10 loop, this region may be a common determinant of substrate specificity in the ADAMTS family. Data from the 756-765/828-835 variant showed that replacing both the β3-β4 and β9-β10 loops had no additional impact on versicanase activity as compared to variant 828-835. It is possible that individual residues in these substitutions may have repulsive effects, thus having a disproportionate effect on versicanase activity. However, according to the AlphaFold model, the β3-β4 and β9-β10 loops compose a single interface (Fig. 6*A*). Therefore, it is possible that once interaction with versican is disrupted by replacing one of these loops, the other one is not able to compensate. The third exosite identified in this study, K795 in loop β6-β7, lies on a different interface (Fig. 6*A*). Since mutations in each of these three loops resulted in decreased versicanase activity (5/10-fold) close to that resulting from the deletion of the Sp (16-fold), it is likely that variants combining mutations in the three loops, if secreted, will fully mimic the versicanase activity of the MDTC variant. However, a caveat when analyzing ADAMTS1 variants with severely decreased versicanase activity is that complete cleavage could not be achieved, even upon prolonged (8 h) incubation time, therefore the computed *k*_cat_/*K*_m_ value may be overestimated.

Following the identification of exosites in ADAMTS4 and ADAMTS5 Sp and Dis domains (31, 75), the data reported here contributes to build up a consensus for proteoglycan-binding exosites in the ADAMTS family. These sequences are composed of short, spaced, positively charged residues, and their identification will make ADAMTS1, like ADAMTS5 (75), amenable to selective targeting by small molecules or biological agents in contexts where excess proteolytic activity may be detrimental such as cancer (18). However, we have also demonstrated that the CysR is critical for ADAMTS1 proteoglycanase activity, therefore future studies should aim to identify exosites in this domain.

To summarize, we have elucidated the functional determinants of ADAMTS1 proteoglycanase activity and identified exosites in the Sp domain. By providing a mechanistic treatment of ADAMTS1 versicanase activity, these findings expand our understanding of the interactions between ADAMTS family members and proteoglycans.

## Experimental procedures

### Generation of ADAMTS1 constructs

The constructs coding for human WT ADAMTS1 with a C-terminal FLAG tag in pCEP4 vector was previously described (31). ADAMTS1 domain-deletion variants were similarly cloned into the pCEP4 vector in-frame with a C-terminal FLAG tag using PCR. The PCR was performed using FL WT ADAMTS1 cDNA as a template and amplified by KOD Hot Start DNA Polymerase (Merck) using forward primer, 5′-ACTGGT*ACCACCATG*CAGCGAGCTGTG-3′ (ADAMTS1 FW) containing a *KpnI* restriction site (underlined) and a Kozak consensus sequence (italic). The following reverse primers, containing the XhoI restriction site (underlined), a stop codon, and the FLAG epitope (in Italics), were used: 5′-CTGCCTCGAGCTA*TTTATCATCATCATCTTTA*-*TAATCGA*AAGATTCCTTCTTCTT-3' (MDTCS); 5′-CTGCCTCGAGCTA*TTTATCATCATCATCTTTATAATCAC*AAGTAGATCCATTTCC-3′ (MDTC); 5′-CTGCCTCGAGCTA*TTTATCATCATCATCTTTATAATCTG*GACAGTCCTCAAGGTT-3' (MDT); 5′- CTGCCTCGAGCTA*TTTATCATCATCATCTTTATAATCAT*GAAAAGGCGTATCAAA-3′ (MD); 5′- CTGCCTCGAGCTA*TTTATCATCATCATCTTTATAATCAG*GCTTGTCCATCAAACA-3′ (M). PCR was carried out for 35 cycles of denaturation (60 s at 94 °C), annealing (60 s at 55 °C), and extension (3 min 30 s at 72 °C). The PCR products were digested with *Kpn*I and *Xho*I (New England Biolabs, 2 h, 37 °C) and ligated into pCEP4 vector using T4 DNA Ligase (16 h, 16 °C).

To generate Sp variants, WT ADAMTS1 was subcloned into pEGFP-N1 vector using *KpnI* and *NotI* restriction enzymes and used as a template for site-directed mutagenesis. PCR products were digested with *DpnI* (New England Biolab Cat. n.: R0176S) to remove the template sequence.

All the constructs were sequenced to confirm that no point mutations were introduced during PCR.

### Expression and purification of recombinant ADAMTS1 variants

ADAMTS1 was expressed and purified as previously reported (31). Human embryonic kidney cells expressing the SV40 large T antigen (HEK293T) were cultured in Minimum Essential Medium Eagle (Sigma-Aldrich) with 10% fetal bovine serum (Labtech), 1 U/ml Penicillin and 0.1 mg/ml Streptomycin (Pen/Strep) (Sigma-Aldrich), 2 mM L-glutamine (Life Technologies), and 1× nonessential amino acids (Sigma-Aldrich) at 37 °C, with 5% CO_2_. After reaching >75% confluence, cells were washed with PBS to remove fetal bovine serum and the medium was replaced with Opti-MEM Gibco (Life Technologies) containing Pen/Strep (1 U/ml and 0.1 mg/ml, respectively) and 2 mM CaCl_2_. Constructs were transiently transfected using PEI (Polysciences Europe GmbH) (PEI/cDNA ratio: 3.6). After 4 h, heparin from porcine intestinal mucosa (Sigma-Aldrich, Cat. n.: H3393, 200 μg/ml) was added to release ADAMTS1 from the ECM. After transfection, cells were incubated for 3 days before harvesting. Protein lysate was extracted using CelLytic M (Sigma-Aldrich Cat. n.: C3228) according to the manufacturer’s instructions.

CM was harvested and centrifuged for 20 min at 1500*g*, followed by filtration (0.45 μm) to remove cell debris and concentrated to 10-fold on a tangential flow filtration system (Millipore) with a 10 kDa cut-off. Purification was performed using *Proteus* 1-step midi spin columns (Generon, Cat. n.: NB-45-00058-2). Medium was incubated with α-FLAG M2 Affinity Gel (Sigma-Aldrich, Cat. n.: A2220), which was pre-equilibrated with TNC-B buffer (20 mM Tris–HCl pH 7.45, 150 mM NaCl, 10 mM CaCl_2_, 0.05% Brij-35), for 2 h at 4 °C. The resin was washed with TNC-B buffer containing 1 M NaCl to remove heparin (32), and bound proteins were eluted with 200 μg/ml FLAG peptide (Sigma-Aldrich, Cat. n.: F3290). The purified samples were then passed through a PD-10 column (GE Healthcare) pre-equilibrated in TNC-B buffer to remove the FLAG peptide. Aliquots taken from each purification step and the purified protein were analyzed using SDS-PAGE, followed by immunoblot and CBB staining. For CBB staining, proteins were loaded based on concentrations measured using optical density and calculated according to the Beer-Lambert law. The following extinction coefficients were used: 109,550 (WT), 92,958 (MDTCS), 79,379 (MDTC), 70,495 (MDT), 67,265 (MD), and 49,025 (M) M^−1^ cm^−1^, respectively. Samples were analyzed under reducing (5% β-mercaptoethanol) and nonreducing conditions on Bolt 4 to 12% Bis-Tris NuPage Gels (Life Technologies).

For immunoblotting, mouse monoclonal anti-FLAG M2 (Cat. n.: F3165 Sigma Aldrich, 1 μg/ml) and anti-actin (Cat. n.: AAN01, Cytoskeleton, UK, 0.5 μg/ml) antibodies were used. Following addition of the appropriate horseradish peroxidase–conjugated antibodies (Agilent Technologies), Immobilon Chemiluminescent HRP substrate (Merck Millipore) was detected with a Chemidoc Touch Imaging system (Bio-Rad), and images were exported using Image lab software version 5.2.1 (Bio-Rad). Purity of protein samples was examined using Imperial Protein Stain (Thermo Fisher Scientific, product code 24615), which is based on the CBB R-250 dye, and washed extensively with distilled water to remove excess dye. Images were taken when proteins bands were clearly visible. Fractions containing pure ADAMTS1 were pooled, concentrated, and stored at −80 °C before activity assays.

### Expression and purification of recombinant proteoglycans and fibulin1

FL versican V1 (42) was purified by anion exchange chromatography as previously described using HiTrap DEAE Sepharose (GE Healthcare) (31). Versican V1-5GAG which is a truncated version of V1, comprising amino acids 21 to 694 of V1 with a C-terminal C-myc/6× His tag, was described previously (31, 42). V1-5GAG was purified according to previously established protocols using nickel affinity purification (31).

Constructs coding for human FL biglycan (UniProt ID P21810) and fibulin1C (UniProt ID P23142-4) with a C-terminal 6x-His tag were custom-synthesized by Invitrogen, cloned into pcDNA 3.1^(+)^ vectors, and transiently transfected into HEK293T cells using PEI as above. Three days posttransfection, the CM was concentrated 15-fold using a Lab scale tangential flow filtration system (Merck) and purified using a Ni-sepharose column (GE Healthcare) previously equilibrated with three column volumes of TBS (20 mM Tris–HCl, pH 7.4, 150 mM NaCl). Following binding, the column was washed with TBS containing 10 mM imidazole and bound proteins were eluted using a linear gradient (10–300 mM) of imidazole. Eluted fractions containing recombinant biglycan were subjected to SDS-PAGE, pooled, concentrated on Amicon Ultra spin columns (10 kDa cut-off), and dialyzed extensively against TBS. Protein concentrations were measured using optical density and calculated according to the Beer-Lambert law using extinction coefficients of 22,6505, 70,540, 38765, and 35,750 M^−1^ cm^−1^ for the protein core of V1, V1-5GAG, biglycan, and fibulin1, respectively, on the Expasy ProtParam web tool.

### Semiquantitative proteoglycan cleavage assays

Purified V1-5GAG (100 nM) was digested with ADAMTS1, in TNC-B buffer at 37 °C for 2 h. Where indicated, 500 μM recombinant human TIMP1, TIMP2, TIMP3, or TIMP4 (R&D Systems, Cat. n.: 970-TM, 971-TM, 973-TM, 974-TSF) were preincubated with 100 nM ADAMTS1 for 1 h at 37 °C before digestion. Biglycan (1130 nM) was digested for 24 h at 37 °C. The reactions were stopped with EDTA (25 mM) in deglycosylation buffer (50 mM sodium acetate, 25 mM Tris–HCl pH 8.0) containing 0.1 U/ml chondroitinase ABC (AMS biotechnology Cat. n: AMS.E1028-02) for 16 h at 37 ºC. Samples were run on SDS-PAGE under reducing conditions and cleavage products were detected by immunoblotting with the following antibodies: goat polyclonal anti-biglycan (R&D Systems, Cat. n. AF2667, 0.4 μg/ml); rabbit polyclonal anti-Vc, recognizing the versican sequence ^432^VPKDPEAAEARRG^445^ which spans the Glu^441^-Ala^442^ cleavage site (1 μg/ml) (42); rabbit polyclonal anti-DPEAAE neoepitope antibody (Life Technologies, Cat. n.: PA1-1748A, 2 μg/ml) which only detects versikine, the N-terminal versican fragment generated after proteolysis at Glu^441^-Ala^442^; goat polyclonal anti-biglycan (R&D Systems, Cat. n.: AF2667, 0.4 μg/ml).

Aggrecan digestion assays were performed as previously described (45). Briefly, purified recombinant ADAMTS1 variants (100 nM) were incubated with bovine nasal aggrecan (purified according to the method of Hascall and Sajdera (76), 330 nM) in TNC-B buffer for 1 h at 37 °C. The reactions were stopped with EDTA buffer, and the samples were deglycosylated in deglycosylation buffer with Chondroitinase ABC and endo-β-galactosidase (each 0.01 unit/100 μg of aggrecan) for 24 h at 37 °C. Aggrecan was then precipitated using ice-cold acetone and analyzed by immunoblotting using either a mouse monoclonal anti-ARGSV neoepitope antibody recognizing aggrecanase cleavage at Glu^392^-Ala^393^ (Life Technologies, Cat n.: MA316888) or a rabbit polyclonal anti-AGEG neoepitope antibody, which recognizes aggrecanase cleavage at Glu^1790^-Ala^1791^ (36). Bands were detected with a Chemidoc Touch Imaging system and intensities were measured using Image lab software version 5.2.1. Sequential exposures were analyzed to avoid saturation artifacts. All quantifications were performed on images taken with the same exposure settings and without postimage processing, with the exception of color thresholding applied equally to all images to reduce background signal.

### Quantitative versican digestion assay

For determination of kinetic constants, ADAMTS1 and its variants (100 nM) were incubated with 50 nM purified versican (V1 or V1-5GAG) for 8 h. Subsamples were removed at different timepoints and reactions were stopped with EDTA in deglycosylation buffer. Following proteolysis, all samples were analyzed by ELISA as before (31). For this, 96-well Maxisorp plates (Nunc) were coated with 5 μg/ml anti-DPEAAE neoepitope antibody in carbonate buffer pH 9.6 at 4 °C for 16 h. Washing steps were performed in triplicate between each step with 300 μl PBS containing 0.1% Tween-20. Samples from the digestion experiments were diluted in 3% BSA/PBS and added to the plate (100 μl, 2 h). Anti-G1 monoclonal antibodies (Abcam, Cat n.: ab171887, 3 μg/ml in 0.5% BSA/PBS) were used to detect bound versikine. The assay was developed by the addition of o-phenylenediamine dihydrochloride (Cat n. 34006, Sigma Aldrich) for 10 min, and reactions were stopped with 2M H_2_SO_4_. The absorbance was read at 492 nm using a BioTeK Epoch (BioTek) plate reader. For each dilution, the amount of versikine generated was derived from a standard curve (0–1.56 nM) of V1-5GAG completely digested with ADAMTS5. Fractional cleavage (y-axis) was plotted against time (x-axis). Specificity constants (*k*_cat_/*K*_m_) were determined in GraphPad Prism using the equation *v*/[E] = [S]*k*_cat_/*K*_m_ as previously described (77).

### QF peptide cleavage assay

QF peptide cleavage assays were conducted in 384-well plates (Cat. n.: 784900, Greiner Bio-One, Austria) on a SpectraMax i3 Multi-Mode Platform (Molecular Devices). The fluorescent peptide fluorescein-5(6)-carbonyl-Ala-Glu-Leu-Asn-Gly-Arg-Pro-Ile-Ser-Ile-Ala-Lys (5(6)-TAMRA) (custom-synthesized by Bachem) was dissolved as a 10 mM stock in dimethylsulfoxide and used at a final concentration of 3.5 μM. QF reactions were performed in a total volume of 20 μl at 37 °C. Fluorescent intensity was recorded with an excitation wavelength of 485 nm and an emission wavelength of 538 nm every min for 2 h, expressed as relative fluorescence units and normalized against a blank containing only buffer and substrate. Titrations were performed as before (39, 54). ADAMTS1 variants were incubated with TIMP3 (0–16 nM) for 1 h at 37 °C before addition of the QF substrate. Relative fluorescence unit values were converted into residual activity by fixing as 100% the activity of the reactions not containing TIMP3. Following linear fitting of the initial portion of the curve, the active site concentration of ADAMTS1 variants was determined by interpolating the value on the x axis, representing the TIMP3 concentration.

### Statistical analysis

Data are presented as mean ± SD of at least three independent experiments and were analyzed by GraphPad Prism Software. Statistical analysis was performed by two-tailed Mann-Whitney tests. *p* < 0.05 was considered significant.

## Data availability

All data described in this study are contained within the manuscript.

## Supporting information

This article contains supporting information (Figs. S1-S5 and Reference (78)).

## Conflict of interest

The authors declare that they have no conflicts of interest with the contents of this article.
