# Supplementary material for: The C-terminal domains of ADAMTS1 contain exosites involved in its proteoglycanase activity
Source: J Biol Chem. 2023 Feb 21;299(4):103048. doi: 10.1016/j.jbc.2023.103048 (PMC10033314; doi:10.1016/j.jbc.2023.103048)

**SUPPORTING INFORMATION**

**The C-terminal domains of ADAMTS1 contain exosites involved in its proteoglycanase activity**

Alexander Frederick Minns,^1^ Yawei Qi,^2^ Kazuhiro Yamamoto,^3^ Karen Lee,^2^ Josefin Ahnström,^2^ and Salvatore Santamaria^1,^*

^1^Department of Biochemical Sciences, School of Biosciences, Faculty of Health and Medical Sciences, Edward Jenner Building, University of Surrey, Guildford, Surrey GU2 7XH, United Kingdom; ^2^Department of Immunology and Inflammation, Imperial College London, Du Cane Road, W12 0NN, London, United Kingdom; ^3^Institute of Life Course and Medical Sciences, University of Liverpool, Liverpool L7 8TX, United Kingdom.

* to whom the correspondence should be addressed: Salvatore Santamaria, Department of Biochemical Sciences, School of Biosciences, Faculty of Health and Medical Sciences, Edward Jenner Building, University of Surrey, Guildford, Surrey GU2 7XH, United Kingdom; [s.santamaria@surrey.ac.uk](mailto:s.santamaria@surrey.ac.uk)

**Running title:** *Determinants of ADAMTS1 proteoglycanase activity*

**Figure S1: CBB stain of ADAMTS1 M under non-reducing conditions.** CBB staining under non-reducing conditions of purified ADAMTS1 M. Red stars indicate the zymogens, white stars differentially *N*-linked glycosylated mature forms, black stars aggregated forms.

**
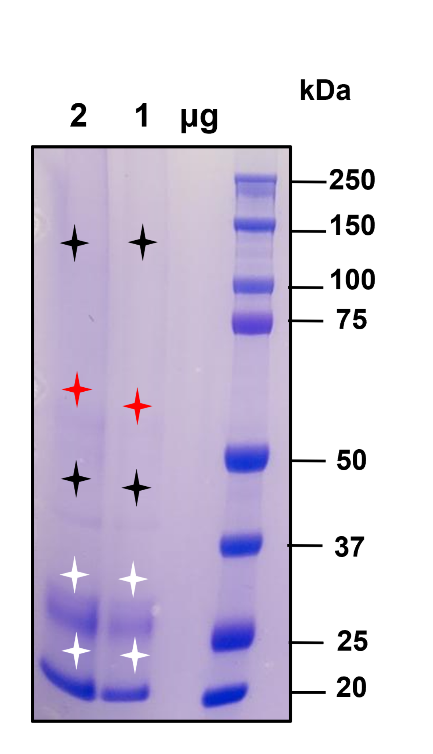
**

**Figure S2:** **Aggrecanase activity of ADAMTS1 compared to ADAMTS4 and ADAMTS5.** Bovine aggrecan (330 nM) was incubated with different concentrations of ADAMTS1, ADAMTS4 and ADAMTS5 (2 h, 37°C). Samples were deglycosylated, subjected to SDS-PAGE and detected using anti-ARGSV neoepitope antibody, which specifically detects cleavage at Glu^392^-Ala^393^. The red arrow indicates the major aggrecanase cleavage fragment generated by cleavage at Glu^392^-Ala^393^ and Glu^1499^-Gly^1500^ (UniProt ID P13608-1), while the blue arrows indicate further degradation products, as previously shown **[45]**. Representative immunoblot (IB) of two independent replicates.

**
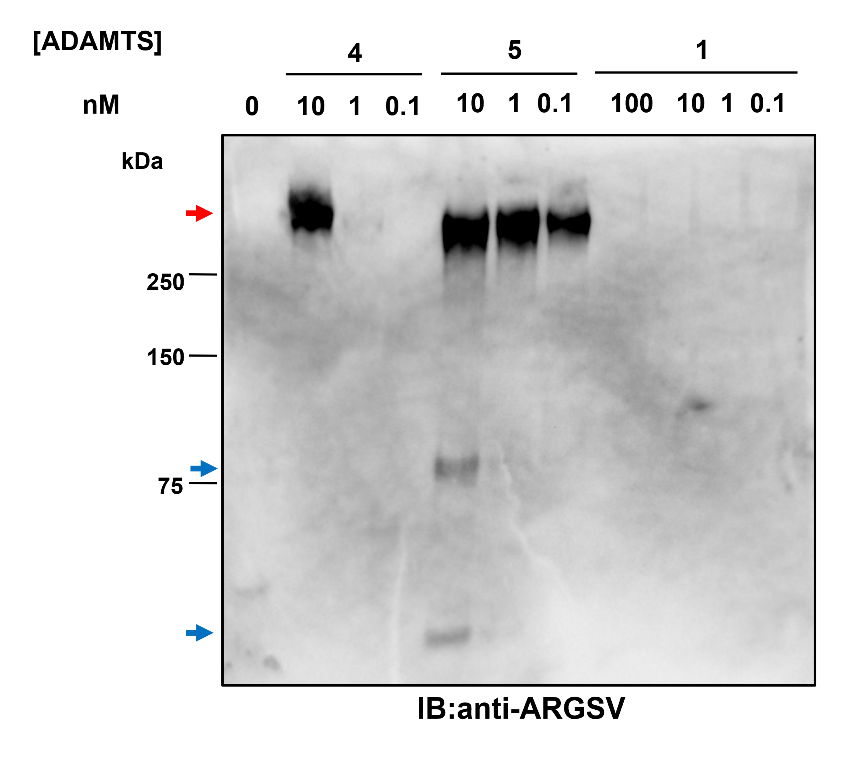
**

**Figure S3. Expression of ADAMTS1 Sp variants.** ADAMTS1 variants were transfected in HEK293T cells. Heparin (200 µg/mL) was added 4 h post-transfection to release ECM- bound forms. Conditioned media (**A**) and cell lysates (**B**) were analyzed by immunoblot using anti-FLAG antibody. Actin was used as a loading control. Red stars indicate the zymogens, blue stars cleavage products. Representative immunoblot (IB) of two independent replicates.


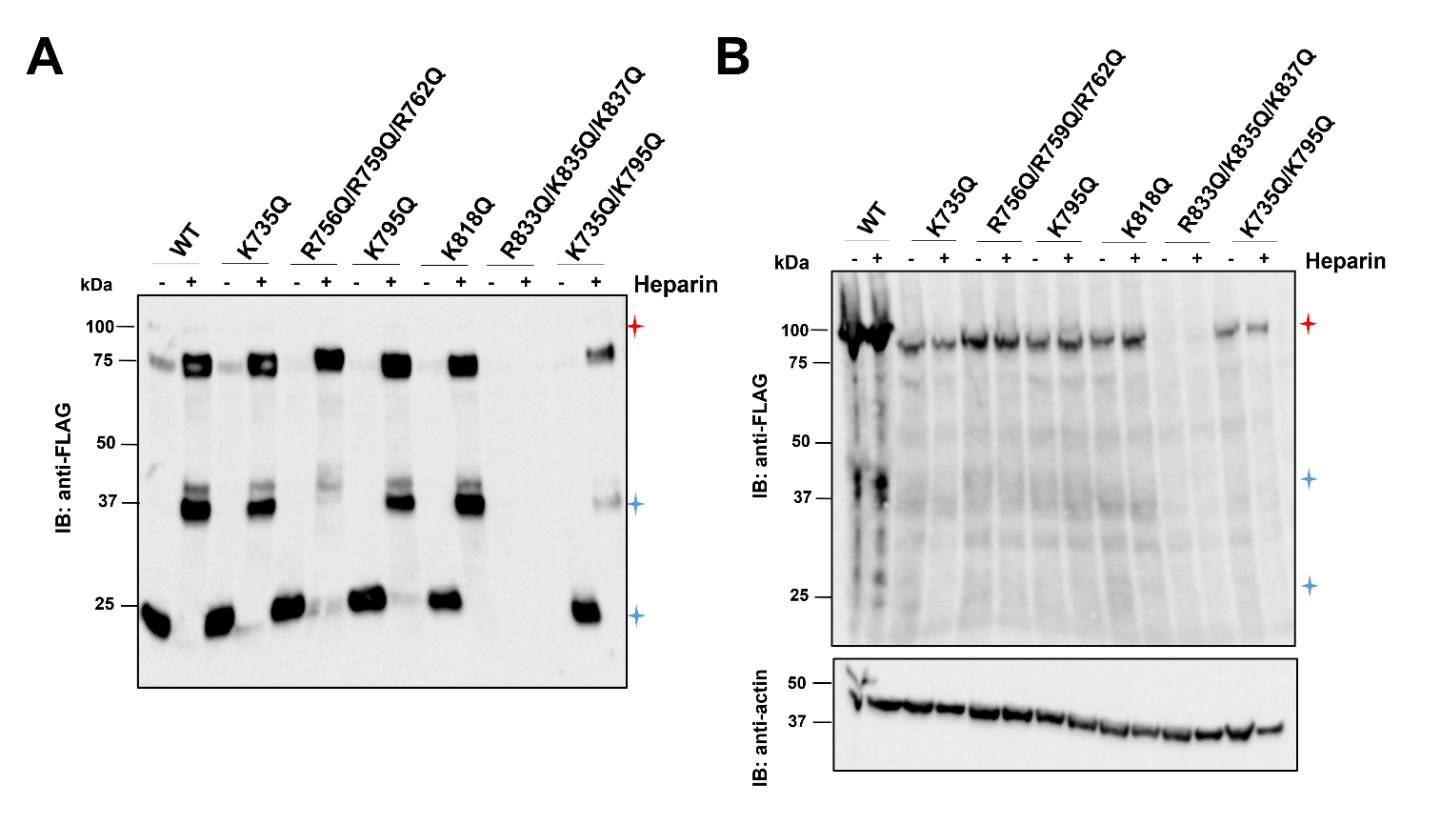


**Figure S4. Sequence alignment of the Sp domain of human and mouse ADAMTS1.** Alignment was performed in Clustal Omega (<https://www.ebi.ac.uk/Tools/msa/clustalo/>) and visualized using MView (<https://www.ebi.ac.uk/Tools/msa/mview/>). UniProt accession numbers were Q9UHI8 for human ADAMTS1 (hATS1, aa 725–749) and P97857 for mouse ADAMTS1 (mATS1, aa 726-850), respectively. Beta strands and interconnecting loops are indicated. Exosite residues are highlighted by red rectangles. Conserved amino acids are colored according to physicochemical properties (purple, positively charged; yellow, negatively charged; green, apolar; cyan, polar). Black asterisks indicate residues with different physcochemical properties, while red asterisks indicate exosites reported in this study.

**
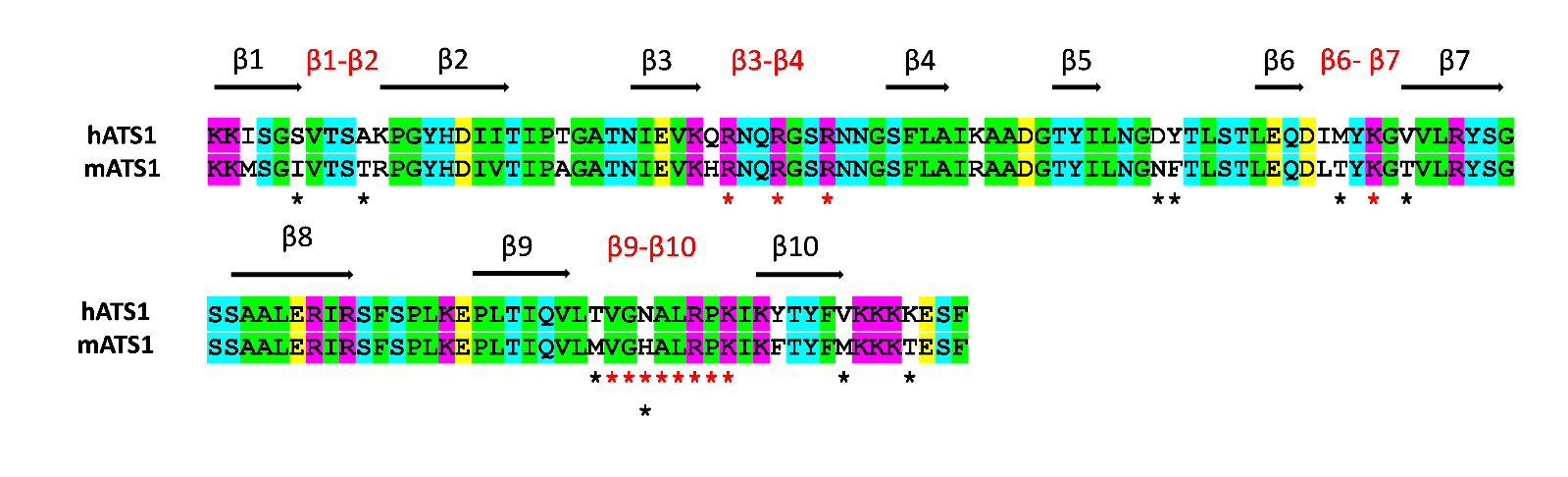
**

**Figure S5. A)** Crystal structure of ADAMTS1 MD (UniProt ID 2JIH). The Mp domain is shown in gold, the Dis domain in green. **B)** Crystal structure of the Mp (UniProt ID 2JIH) colored according to the hydrophocity index **[78]**, with the most hydrophobic residues in red. The molecule has the same orientation as in panel A. Models were visualized using PyMOL.


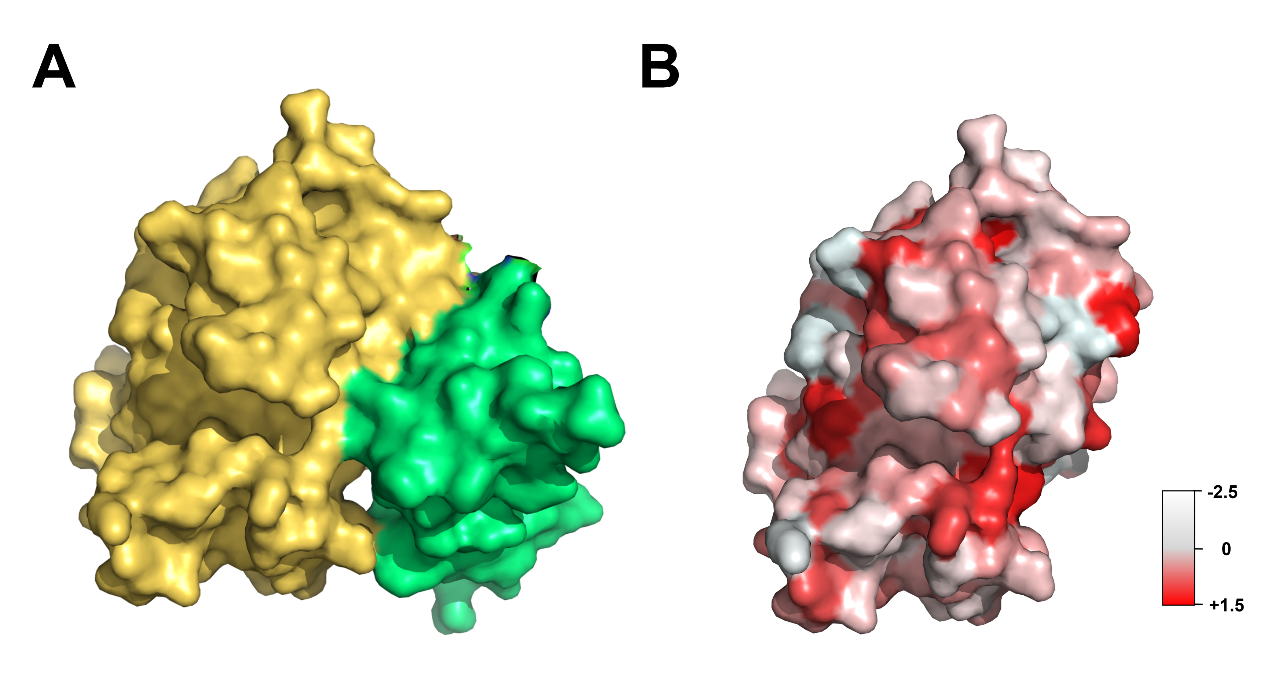

Supplement: Supporting information [file mmc1.docx]
